# Comparative Characterization of Fruit Volatiles and Volatile-Related Genes Expression of ‘Benihoppe’ Strawberry and Its Somaclonal Mutant

**DOI:** 10.3390/plants12051109

**Published:** 2023-03-01

**Authors:** Zhuo Zhang, Shuang Yu, Zhihong Zhang, Junxiang Zhang, He Li

**Affiliations:** 1 Liaoning Key Laboratory of Strawberry Breeding and Cultivation, College of Horticulture, Shenyang Agricultural University, Shenyang 110866, China; 2 Laboratory of Protected Horticulture (Shenyang Agricultural University), Ministry of Education, Shenyang 110866, China; 3 Analytical and Testing Center, Shenyang Agricultural University, Shenyang 110866, China

**Keywords:** *Fragaria* × *ananassa*, somaclonal variation, volatile compounds, gene expression

## Abstract

Somaclonal variations in tissue cultures can be used in plant breeding programs. However, it is still unclear whether somaclonal variations and their original parent have differences in volatile compounds, and the candidate genes which result in the differences in volatile compounds also need to be identified. In this study, we utilized the ‘Benihoppe’ strawberry and its somaclonal mutant ‘Xiaobai’, which has different fruit aromas compared with ‘Benihoppe’, as research materials. Using HS-SPME-GC-MS, 113 volatile compounds have been identified in the four developmental periods of ‘Benihoppe’ and ‘Xiaobai’. Among them, the quantity and content of some unique esters in ‘Xiaobai’ were much higher than that in ‘Benihoppe’. In addition, we found that the contents and odor activity values of ethyl isovalerate, ethyl hexanoate, ethyl butyrate, ethyl pentanoate, linalool, and nerolidol in the red fruit of ‘Xiaobai’ were much higher compared with ‘Benihoppe’, which may result from the significantly increased expression of *FaLOX6*, *FaHPL*, *FaADH*, *FaAAT*, *FaAAT1*, *FaDXS*, *FaMCS*, and *FaHDR* in ‘Xiaobai’. However, the content of eugenol in ‘Benihoppe’ was higher than that in ‘Xiaobai’, which may result from the higher expression of *FaEGS1a* in ‘Benihoppe’ compared with ‘Xiaobai’. The results provide insights into the somaclonal variations that affect the volatile compounds in strawberries and can be used for strawberry quality improvement.

## 1. Introduction

Strawberries belong to the Rosaceae family. Strawberry fruits with pleasant aromas are popular with consumers all over the world [1]. The volatile aroma is one of the most important features of strawberries. The aromas of strawberries are typically complex mixtures, which include esters, furanones, terpenoids, lactones, aldehydes, alcohols, and sulfur compounds [2,3]. The biosynthetic pathways of aromas mainly include the amino acid pathway, terpenoid pathway, fatty acid pathway, and carbohydrate pathway [4,5]. To date, more than 360 volatile aroma components have been isolated and identified in strawberries [6]. A significant negative correlation has been found between the threshold and intensity of the aroma [7]. The odor activity value (OAV) is a good indicator of key aroma components [8]. The OAV of volatile components makes a great contribution to the strawberry’s flavor only when the value is greater than one [9]. In addition, some aroma components have lower sensory thresholds despite higher concentrations, while some volatile compounds have a significant effect on the characteristic volatile compounds of strawberries at lower levels, such as sulfur [8].

Previous research has illustrated that esters account for 25~90% of total volatile components in most strawberry cultivars [10]. Methyl ester and ethyl ester significantly accumulate in ripening fruits [10]. The biosynthesis of esters mainly results from the fatty acid pathway [11] and the amino acid pathway [12]. Linear esters are mostly synthesized by the lipoxygenase or the β-oxidation pathways, whereas most branched-chain esters are derived from the degradation of branched-chain amino acids [13]. Lactones are cyclic esters with peach-like aromas, and are prominent volatile compounds in some plant species [14], such as peach [15] and strawberry [16,17]. Both γ-decalactone and γ-dodecalactone have sweet-enhancing effects, whereas only γ-dodecalactone plays an important role in taste [18]. Aldehydes and furans also play important roles in strawberry flavor, and they account for 50% of the total volatile compounds [19]. Terpenoids and sulfur compounds [20] make up less than 10% and 2% of the total volatile compounds, respectively. They play an important role in the aroma characteristics of strawberries. Alcohols make up 35% of the total volatile compounds, but they have little effect on strawberry flavor [21]. These results indicate that different aroma components are synthesized through different metabolic pathways, and different aroma components have different contents, concentrations, and effects on the characteristic volatile compounds in strawberries. The discovery of important genes involved in volatile biosynthesis pathways reveals the molecular mechanism of plant and fruit aroma regulation, such as *HPL*, *ADH*, *AAT*, *TPS*, etc. [22]. In addition, many factors affect the volatile aromas of strawberries, including genetics [23], temperature [24,25], maturity [26], pre-harvest and post-harvest [9]. However, whether the somaclonal mutation of strawberries affects variations in volatile compounds is largely unknown.

Somaclonal disparity originated from tissue culture germinating variations in regenerated plants [27]. The variant with useful agronomic traits is a source for breeding [28]. In this study, we detected the volatile compounds in different developmental stages of ‘Benihoppe’ and its somaclonal mutant ‘Xiaobai’ using HS-SPME-GC-MS. We further analyzed the expression of volatile-related genes in the main volatile metabolic pathways to identify the genes responsible for volatile compound differences between ‘Benihoppe’ and ‘Xiaobai’. An integration of volatile compounds and volatile-related gene expression of the ‘Benihoppe’ strawberry and ‘Xiaobai’ will provide a new vision for the impact of somaclonal mutants on aroma components of strawberries, and will be useful for strawberry quality improvement.

## 2. Results

### 2.1. Comparative Analysis of Phenotypes and Nine Categories of Volatile Compounds in the Fruits of ‘Benihoppe’ and ‘Xiaobai’ at Different Developmental Stages

**Figure 1:**
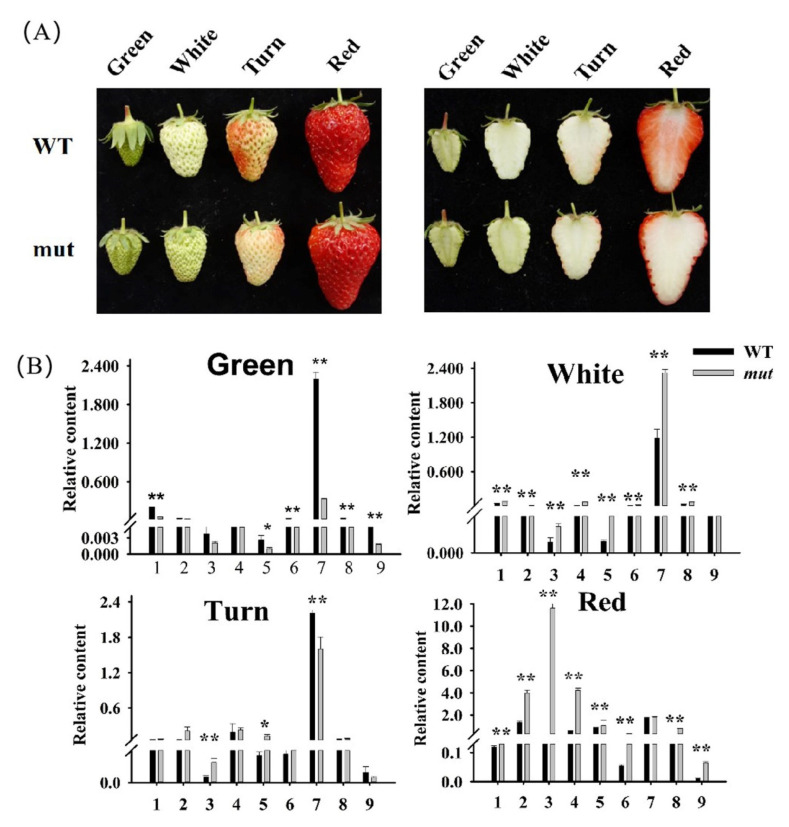
(**A**) Fruit phenotype in four developmental stages of ‘Benihoppe’ and ‘Xiaobai’; (**B**) Volatile compounds relative content in four developmental stages of ‘Benihoppe’ and ‘Xiaobai’; 1-9: Alcohol, acid, ethyl ester, acetate ester, other esters, benzene and other volatile phenols, aldehydes and ketones, isoprenoids, furan. (WT: ‘Benihoppe’; mut: ‘Xiaobai’). (*, *p* < 0.05; **, *p* < 0.01).

We first compared the phenotypes of ‘Benihoppe’ (WT) and ‘Xiaobai’ (mut) strawberry fruit at four different developmental stages. The fruit skin and flesh color of ‘Benihoppe’ and ‘Xiaobai’ showed no significant differences at the green fruit, white fruit and turning fruit stages. During the ripening fruit stage, ‘Benihoppe’ had red skin and red flesh, whereas ‘Xiaobai’ had red skin and white flesh (Figure 1A). To investigate whether the volatile compounds found in ‘Benihoppe’ and ‘Xiaobai’ were also different, we tested volatile compounds in the four developmental stages of ‘Benihoppe’ and ‘Xiaobai’ fruit using HS-SPME-GC-MS. A total of 113 volatile compounds were detected in the four developmental stages of ‘Benihoppe’ and ‘Xiaobai’ fruit (Tables S2 and S3).

We further analyzed the relative abundance of the volatile compounds in the four developmental stages of ‘Benihoppe’ and ‘Xiaobai’ fruit (Figure 1B). We found the highest relative abundances were aldehydes and ketones in the green fruit, white fruit, and turning fruit stages (Figure 1B). During the red fruit stage, the relative accumulation of aldehydes and ketones gradually decreased, whereas the relative accumulation of esters gradually increased (Figure 1B). The relative contents of acids, ethyl esters, acetates esters, other esters, benzene and volatile phenols, isoprenoids, and furan gradually increased in ‘Benihoppe’ and ‘Xiaobai’ during fruit ripening (Figure 1B). In the red fruit stage, the relative contents of acids (2.96-fold), ethyl esters (56.57-fold), acetates esters (7.29-fold), other esters (1.23-fold), aldehydes and ketones (1.04-fold), alcohols (1.12-fold), benzene and volatile phenols (5.93-fold), isoprenoids (3.60-fold), and furan (5.99-fold) in the ‘Xiaobai’ were significantly higher than those in ‘Benihoppe’ strawberry fruit (Table S5). These results indicated that the contents of the volatile compounds, except for alcohols, aldehydes, and ketones, were dramatically increased in the red fruit of ‘Xiaobai’ compared with ‘Benihoppe’. In the red fruit stage, the aldehydes and ketones accounted for 33.74% of the total volatile compounds and the esters (including the ethyl esters, acetates esters, and other esters) accounted for 32.24% of the total volatile compounds in ‘Benihoppe’ (Table S4). However, the aldehydes and ketones accounted for only 7.52% of the total volatile compounds and the esters (including the ethyl esters, acetates esters, and other esters) accounted for 70.45% of the total volatile compounds in the red fruit of ‘Xiaobai’ (Table S4). Therefore, in the red fruit stage, the contents of esters and the percentages of esters in total volatile compounds in ‘Xiaobai’ were much higher than those in ‘Benihoppe’.

### 2.2. Comparative Analysis of 113 Volatile Compounds in the Fruits of ‘Benihoppe’ and ‘Xiaobai’ at Different Developmental Stages

**Figure 2:**
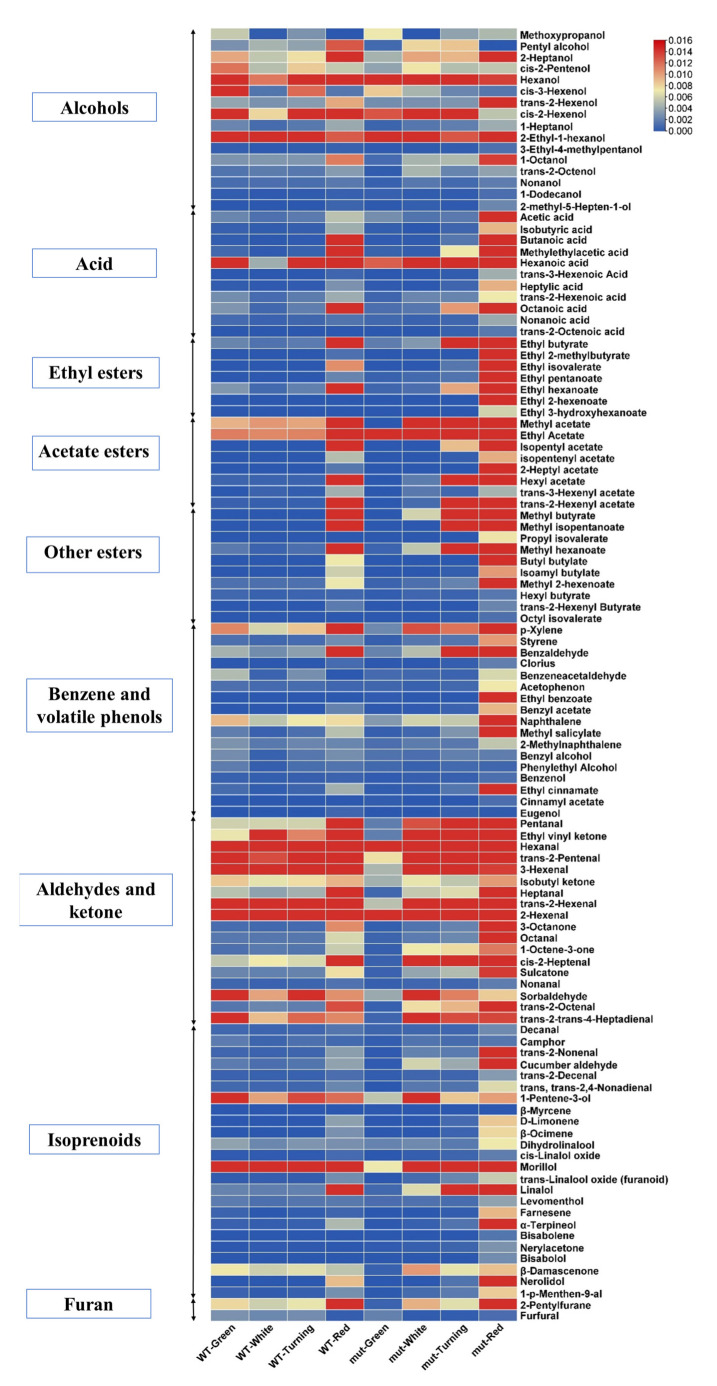
Heatmaps of volatile compounds in different developmental stages of ‘Benihoppe’ and ‘Xiaobai’. Darker red indicates a higher compound content. Darker blue indicates a lower compound content. (WT: ‘Benihoppe’; mut: ‘Xiaobai’).

Different ratios of volatile substances often determine aroma properties. In the fruit of ‘Benihoppe’ and ‘Xiaobai’, 113 compounds were identified (Figure 2). We found that the contents of alcohols and aldehydes were similar in ‘Benihoppe’ and ‘Xiaobai’ across the four developmental stages. Interestingly, the red fruit of ‘Xiaobai’ had higher concentrations of acetic acid, ethyl 2-methylbutyrate, ethyl hexanoate, ethyl butyrate, ethyl pentanoate, ethyl 2-hexenoate, 2-heptyl acetate, propyl isovalerate, methyl 2-hexenoate, ethyl benzoate, benzyl acetate, naphthalene, methyl salicylate, ethyl cinnamate, trans-2-nonenal, linalool, farnesene, α-terpineol, and nerolidol, whereas the contents of these substances were very low in the red fruit of ‘Benihoppe’ (Figure 2).

### 2.3. Comparative Analysis of Unique Volatile Compounds in ‘Benihoppe’ and ‘Xiaobai’

To further compare the differences in volatile compounds between ‘Benihoppe’ and ‘Xiaobai’, the unique volatile compounds in the fruit of ‘Benihoppe’ and ‘Xiaobai’ at four developmental periods were compared. As shown in Tables S6 and S7, 12 unique volatile compounds were identified in ‘Benihoppe’, whereas 35 unique volatile compounds were identified in ‘Xiaobai’. In the red fruit stage, there are 12 unique volatile compounds in ‘Xiaobai’, including 1 alcohol, 1 acid and 4 esters, 3 benzene and volatile phenols, and 3 isoprenoids (Table S7), whereas only 1 unique volatile compound was identified in ‘Benihoppe’ (Table S6). These results illustrated that the amounts of unique volatile compounds were much higher in the turning and red fruit stage of ‘Xiaobai’ than those in ‘Benihoppe’, which may be one of the reasons for the difference in aromas between ‘Benihoppe’ and ‘Xiaobai’.

### 2.4. The OAVs of the Main Volatile Compounds in ‘Benihoppe’ and ‘Xiaobai’

The OAVs of 30 volatile compounds in ‘Benihoppe’ and ‘Xiaobai’ fruit across four developmental stages were calculated based on the threshold values which have been reported in strawberries [2,7,8,14,29,30]. A total of 13 and 10 volatile compounds with OAVs greater than 1 in the red fruit were identified in ‘Xiaobai’ and ‘Benihoppe’, respectively (Table 1). In addition, 17 and 20 volatile compounds with OAVs less than 1 in red fruit were identified in ‘Xiaobai’ and ‘Benihoppe’, respectively (Table 1). Among them, the OAVs of ethyl isovalerate, ethyl hexanoate, ethyl butyrate, ethyl pentanoate, and linalool in ‘Xiaobai’ were 146.5, 86.4, 34.6, 24.9, and 3.6 times higher than those in ‘Benihoppe’, respectively. Therefore, the OAVs of these characteristic aroma compounds in ‘Xiaobai’ were much higher than those in ‘Benihoppe’, which illustrated that these volatile compounds had an important contribution to the aroma of ‘Xiaobai’. In addition, in the red fruit stage, the OAVs of ethyl acetate and nerolidol in ‘Xiaobai’ were greater than one, whereas the OAVs of these two components in ‘Benihoppe’ were less than one, which indicated that ethyl acetate and nerolidol also contributed to the aroma of ‘Xiaobai’.

### 2.5. Principal Component Analysis of Volatile Compounds in ‘Benihoppe’ and ‘Xiaobai’

**Figure 3:**
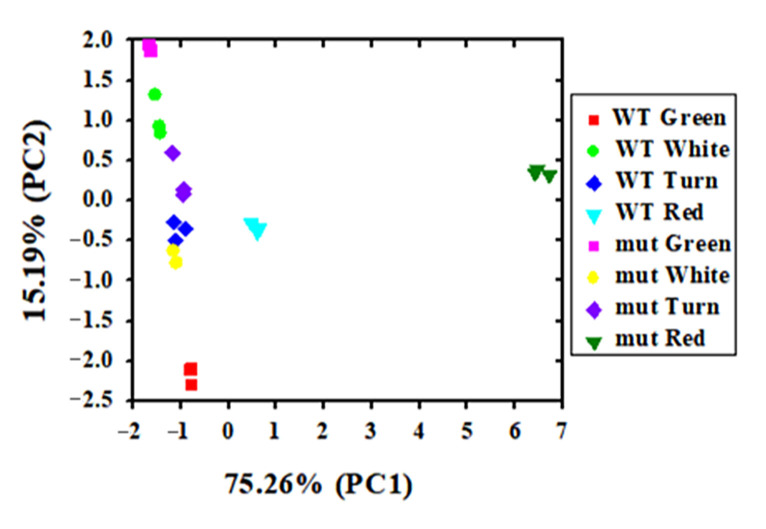
Principal component analysis of relative contents of nine categories of volatile compounds in ‘Benihoppe’ and ‘Xiaobai’ (WT: ‘Benihoppe’; mut: ‘Xiaobai’).

To detect the changing trend of the relative contents of volatile substances in the fruit of ‘Benihoppe’ and ‘Xiaobai’ in four developmental stages, principal component analysis was conducted based on the content of nine categories of volatile substances. The first principal component (PC1) accounted for 75.26% of the total variance, and the second principal component (PC2) accounted for 15.19%. PC1 and PC2 successfully distinguished the contents of volatile compounds in ‘Benihoppe’ and ‘Xiaobai’ at four developmental stages (Figure 3). There are also obvious differences in the relative contents of volatile compounds between ‘Benihoppe’ and ‘Xiaobai’ in the same fruit development period (Figure 3). Based on the principal component loading matrix of the volatile compounds in Table S8, we found that the contribution rates of volatile compounds under PC1 from largest to smallest were isoprenoids, benzene and volatile phenols, acetates esters, acids, furans, ethyl esters, other esters, alcohols, aldehydes, and ketones. Together, these results showed that significant differences in the contents of volatile compounds between ‘Benihoppe’ and ‘Xiaobai’ were found at four fruit developmental stages.

### 2.6. Expression Analysis of Genes Related to Volatile Compounds Metabolic Pathway

In order to understand why the volatile compounds were different between ‘Benihoppe’ and ‘Xiaobai’, we examined the correlation between the transcript abundance of volatile-related genes and the contents of volatile compounds in ‘Benihoppe’ and ‘Xiaobai’ at four different developmental stages.

#### 2.6.1. Fatty Acid Pathway

**Figure 4:**
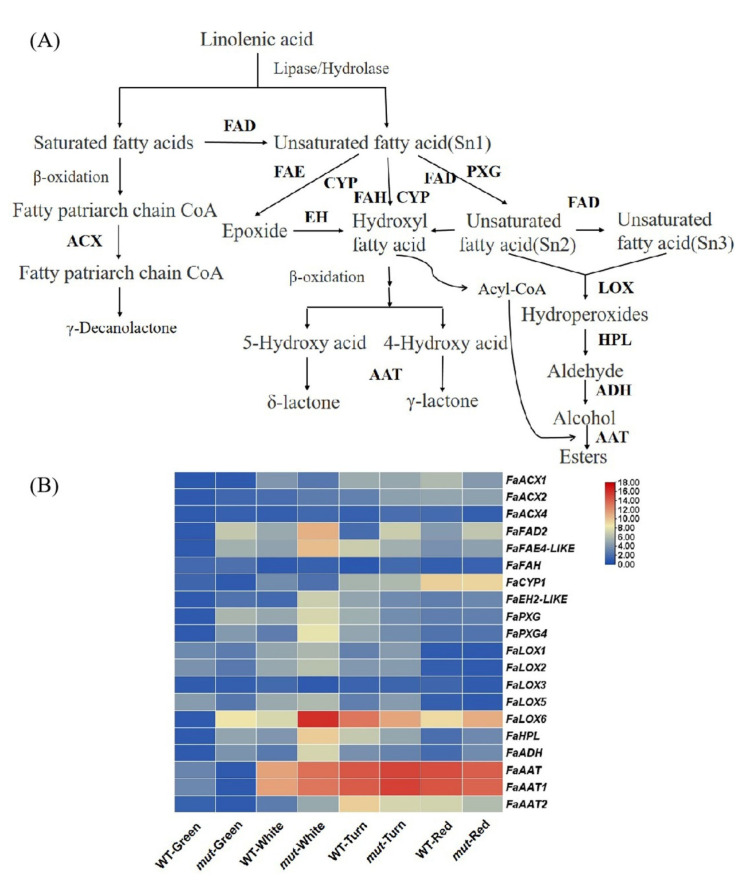
Fatty acid pathway analysis. (**A**) Schematic diagram of the fatty acid pathway. Multiple enzymatic reactions are illustrated by stacked arrows. Abbreviations: ACX: acyl-CoA oxidases; FAD: fatty acid desaturase; FAE: fatty acid elongase; HPL: hydroperoxide lyase; CYP: cytochrome P450; FAH: fatty acid hydroxylase; PXG: peroxygenase; EH: epoxide hydrolase; LOX:lipoxygenase; ADH: alcohol dehydrogenases; AAT: alcohol acyl-transferases; (**B**) Expression levels of biosynthetic genes related to the fatty acid pathway of the ‘Benihoppe’ strawberry (WT) and its ‘Xiaobai’ (mut) fruit at four developmental stages. Darker red indicates a higher expression level, whereas darker blue indicates a lower expression level.

The aroma volatiles of fruit are mainly synthesized through the fatty acid pathway; this pathway can produce alcohols, aldehydes, esters, and lactones. This pathway contains the lipoxygenase (LOX) and β-oxidation pathways. For the LOX pathway, linoleic acid or linolenic acid is first oxidized to hydroperoxide by LOX, which is subsequently cleaved by hydroperoxide lyase (HPL) to form hexanal and hexenal. The C6 aldehyde is reduced to the corresponding C6 alcohol by alcohol dehydrogenase (ADH), then converted to ester by alcohol acyl-transferases (AAT) [31,32] (Figure 4A).

In this study, we found that the content of esters in ‘Xiaobai’ was higher than that in ‘Benihoppe’ in the red fruit stage. There were significant differences in the expression levels of *FaLOX6*, *FaHPL*, *FaADH*, *FaAAT*, and *FaAAT1* genes in the fatty acid pathway in the fruit of ‘Benihoppe’ and ‘Xiaobai’ at four different developmental stages. During the fruit development of ‘Benihoppe’ and ‘Xiaobai’, the expression level of *FaLOX6, FaHPL,* and *FaADH* first increased and then decreased. However, the expression levels of these genes reached the highest values in the turning fruit stage of ‘Benihoppe’ and the white fruit stage of ‘Xiaobai’. In addition, the content of hexanal in ‘Xiaobai’ also first increased and then decreased (Table S3). The content of hexanal in ‘Xiaobai’ reached the maximum in the white fruit stage compared with other fruit development stages, which was consistent with the expression of *FaHPL* in ‘Xiaobai’ (Figure 4B). The gene expression of *FaAAT* and *FaAAT1* in ‘Benihoppe’ and ‘Xiaobai’ gradually increased during fruit ripening, which was consistent with the changing trend of esters in ‘Benihoppe’ and ‘Xiaobai’ during fruit ripening (Figure 4B). Interestingly, the expression levels of *FaLOX6, FaHPL,* and *FaADH* in the red fruit stage of ‘Xiaobai’ were 3.6, 3.2, and 4.0 times higher, respectively, than those in ‘Benihoppe’, which was consistent with the significantly increased esters in the red fruit stage of ‘Xiaobai’ compared with ‘Benihoppe’ (Figure 4B).

#### 2.6.2. Amino Acid Pathway

**Figure 5:**
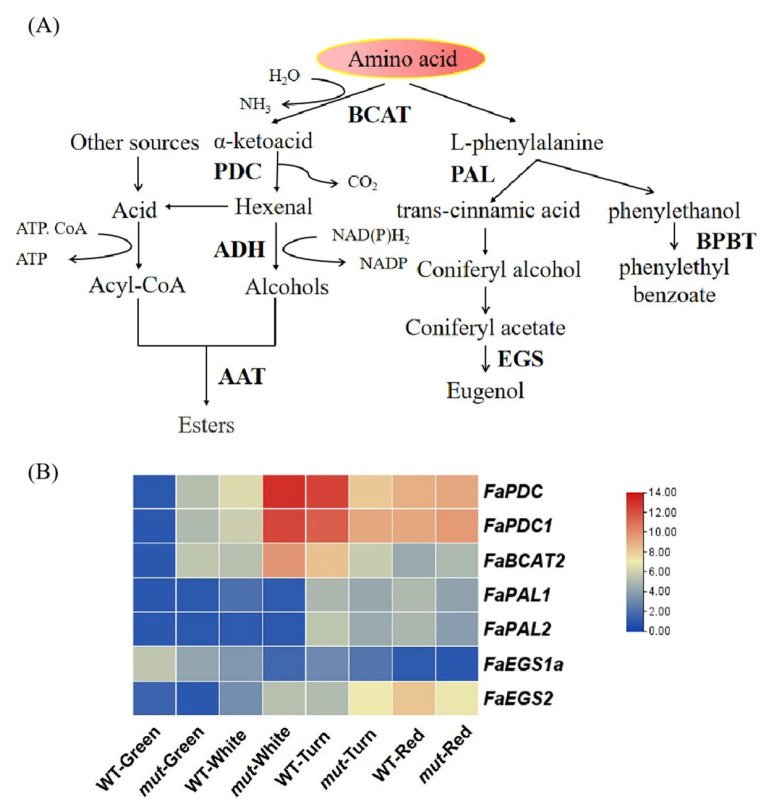
Amino acid pathway analysis. (**A**) Schematic diagram of the amino acid pathway. Multiple enzymatic reactions are illustrated by stacked arrows. Abbreviations: BCAT: branched-chain amino acid transaminase; PDC: pyruvate decarboxylase; ADH: alcohol dehydrogenases; AAT: alcohol acyl-transferases; PAL: phenylalanine ammonia lyase; EGS: eugenol synthase; (**B**) Expression level of biosynthetic genes related to the amino acid pathway in the fruit of ‘Benihoppe’ (WT) and ‘Xiaobai’ (mut) at four developmental stages. Darker red indicates a higher expression level, whereas darker blue indicates a lower expression level.

The amino acid metabolic pathway produces branched-chain alcohols, aldehydes, and acids [12]. Amino acid-derived alcohols and acids can be esterified into compounds that have a greater impact on fruit aromas, such as 3-methyl-butyl acetate and 3-methyl butyrate in bananas [33]. Aromatic amino acids such as phenylalanine, utilize phenylalanine as the initial substrate, which undergoes a deamination reaction under the catalytic action of phenylalanine lyase (PAL) to generate trans-cinnamic acid [34]. Trans-cinnamic acid produces eugenol through a series of enzymes [35] (Figure 5A).

The transcript abundances of genes involved in the amino acid pathway were analyzed in ‘Benihoppe’ and ‘Xiaobai’ at four developmental stages of fruit. The highest expression levels of *FaBCAT2, FaPDC, FaPDC1,* and *FaADH* (Figure 4B) were identified in the white fruit stage of ‘Xiaobai’, but in the turning fruit stage of ‘Benihoppe’. The expression levels *FaAAT* and *FaAAT1* gradually increased during fruit ripening, and were similar between ‘Benihoppe’ and ‘Xiaobai’ (Figure 5B). In addition, the content of eugenol in ‘Benihoppe’ was higher than that in ‘Xiaobai’ (Tables S2 and S3). To explore the reason for the difference in eugenol content between ‘Benihoppe’ and ‘Xiaobai’, we analyzed the expression levels of *FaEGS1a* and *FaEGS2*, which were the key genes that participated in eugenol biosynthesis [36]. We found that there was an opposite expression pattern of *FaEGS1a* and *FaEGS2* in ‘Benihoppe’ and ‘Xiaobai’. The expression of *FaEGS1a* was higher in the green fruit stage, and its expression significantly decreased during fruit ripening. In the four developmental stages, the expression of *FaEGS1a* in ‘Benihoppe’ was higher than that in ‘Xiaobai’. The expression levels of *FaEGS1a* in ‘Benihoppe’ were 2.64, 5.38, 1.93, and 1.15 times higher than those in ‘Xiaobai’, respectively. The gene expression level of *FaEGS2* significantly increased during fruit ripening, and its expression in ‘Benihoppe’ was higher than that in ‘Xiaobai’ in the red fruit stage. The transcript level of *FaEGS2* in ‘Benihoppe’ was 2.5 times higher than that in ‘Xiaobai’ (Figure 5B). Therefore, the higher expression of *FaEGS1a* and *FaEGS2* in ‘Benihoppe’ compared with in ‘Xiaobai’ may lead to a higher content of eugenol in ‘Benihoppe’ than in ‘Xiaobai’, which may be one of the reasons for the difference in aroma between ‘Benihoppe’ and ‘Xiaobai’.

#### 2.6.3. Terpenoid Pathway

**Figure 6:**
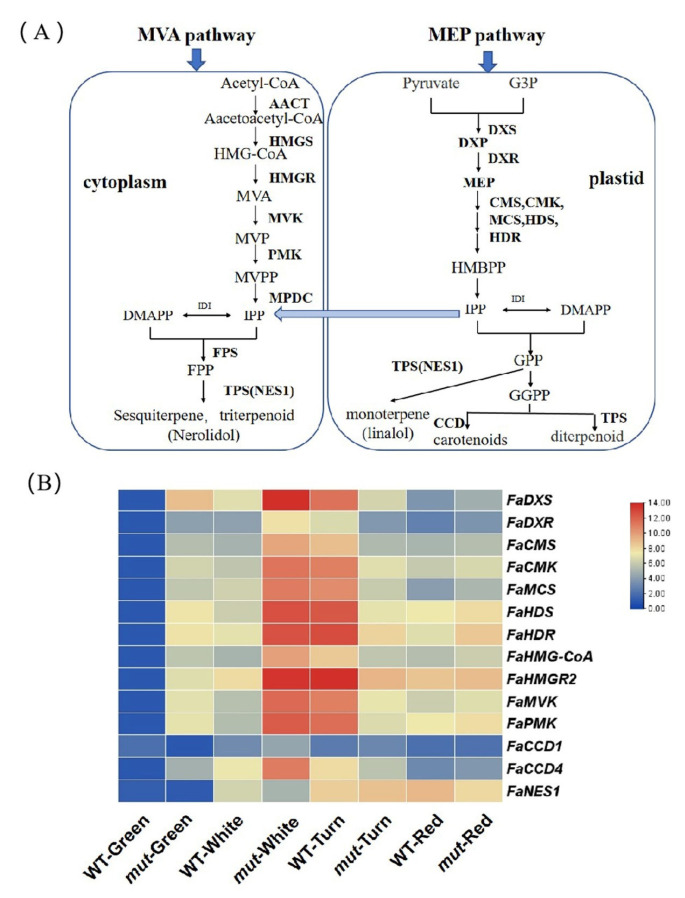
Terpenoid pathway analysis. (**A**) Schematic diagram of terpenoid pathways. Abbreviations: MEP: methylerythritol phosphate; MVA: mevalonic acid; G3P: glyceraldehyde 3-phosphate; DXP: 1-deoxy-D-xylulose-5-phosphate; DXS: DXP synthase; DXR: DXP reductoisomerase; CMS: 2-C-methyl-D-erythritol 4-phosphate cytidylyltransferase; CMK: 4-(cytidine 5′-diphospho)-2-C-methyl-D-erythritol kinase; MCS: 2-C-methyl-D-erythritol 2, 4-cyclodiphosphate synthase; HDS: 4-hydroxy-3-methylbut-2-en-1-yl diphosphate synthase; HDR: HMBPP reductase; IPP: isopentenyl diphosphate; IDI: isopentenyl pyrophosphate isomerase; DMAPP: dimethylallyl diphosphate; FPP: farnesyl diphosphate; GPP: geranyl pyrophosphate; GGPP: geranylgeranyl pyrophosphate; AACT: acetyl-CoA acetyltransferase; HMGS: HMG-CoA synthase; HMGR: HMG-CoA reductase; PMK: phosphomevalonate kinase; MPDC: mevalonate diphosphate decarboxylase; NES1: nerolidol synthase 1; CCD: carotenoid cleavage dioxygenases; (**B**) Transcript level of biosynthetic genes related to terpenoid pathways in the fruit of ‘Benihoppe’ (WT) and ‘Xiaobai’ (mut) at four developmental stages. Darker red indicates a higher expression level, whereas darker blue indicates a lower expression level.

Terpenoids are important volatile compounds that play important roles in the formation of aromas [37,38]. Terpenoids are derived from the common precursor isopentenyl pyrophosphate (IPP) and its allylic isomer, dimethylallyl diphosphate (DMAPP), which are produced by the mevalonic acid (MVA) and methylerythritol phosphate (MEP) pathways, respectively [39]. The MEP pathway operates in the plastids and is mainly responsible for the formation of monoterpenes and diterpenes [40]. The MVA pathway operates in the cytoplasm, endoplasmic reticulum, and peroxisomes, providing precursors for the synthesis of sesquiterpenes (Figure 6A).

Nerolidol and linalool play important roles in the aroma of strawberries. Linalool and nerolidol were generated through *FaNES1* based on geranyl diphosphate (GPP) and farnesyl diphosphate (FPP), respectively [23]. We found that the contents of nerolidol and linalool in ‘Xiaobai’ were higher relative to ‘Benihoppe’. To explore the reason for the difference between nerolidol and linalool in ‘Benihoppe’ and ‘Xiaobai’, we analyzed the genes involved in the terpenoid pathways in the fruit of ‘Benihoppe’ and ‘Xiaobai’ at four developmental stages. We found that the expression levels of *FaDXS, FaDXR*, *FaCMS, FaMCS, FaHDS,* and *FaHDR* in the MVA pathway and *FaHMGR2, FaMVK,* and *FaPMK* in the MEP pathway first increased and then decreased in ‘Benihoppe’ and ‘Xiaobai’. The expression levels of these genes reached their maximum in the turning stage of ‘Benihoppe’ and in the white fruit stage of ‘Xiaobai’. In addition, the transcript abundance of *FaNES1* gradually increased with the ripening of the fruit in ‘Benihoppe’ and ‘Xiaobai’ (Figure 6B). Interestingly, the expression levels of *FaDXS, FaMCS,* and *FaHDR* were significantly increased in the red fruit of ‘Xiaobai’ compared with ‘Benihoppe’, which may result in increased linalool and nerolidol in ‘Xiaobai’.

## 3. Discussion

Somatic variation can be used for plant improvement [41]. In the progeny of micropropagated plants of ‘Benihoppe’, we identified a somaclonal mutant ‘Xiaobai’, which had a special aroma compared to ‘Benihoppe’. To clarify the differences in aroma between ‘Benihoppe’ and ‘Xiaobai’, the main differential components and the differentially expressed genes in ‘Benihoppe’ and ‘Xiaobai’ involved in the pathway of volatile compounds were investigated.

Approximately 360 volatile compounds have been identified in strawberries to date, which mainly include esters, furanones, terpenoids, lactones, aldehydes, alcohols, and sulfur compounds [6]. Significant differences in the aroma compositions of strawberry fruit are related to different cultivars [42,43] and different developmental stages [44]. In this study, we identified 113 volatile compounds in the four developmental periods of ‘Benihoppe’ and ‘Xiaobai’. Volatile compounds were more abundant in ‘Xiaobai’ than in ‘Benihoppe’. Fruit aromas are primarily made up of esters [21]. Our results illustrated that the proportion of esters in the four stages of fruit development in ‘Xiaobai’ was much higher than that in ‘Benihoppe’, especially in the red fruit stage of ‘Xiaobai’ (Table S4). In addition, the OAVs of ethyl isovalerate, ethyl hexanoate, ethyl butyrate, and ethyl pentanoate in ‘Xiaobai’ were much higher than those in ‘Benihoppe’ (Table 1). These results showed that the differences in esters were the key reason for the differences in aroma between ‘Benihoppe’ and ‘Xiaobai’. The formation of aroma is closely related to the expression of aroma-related genes [45]. Ester compounds are mainly synthesized through the fatty acid pathway [11] and the amino acid pathway [12]. Therefore, we examined the expression of genes involved in the fatty acid and amino acid pathways. The expression levels of *FaLOX6*, *FaHPL,* and *FaADH* in the four developmental stages of ‘Xiaobai’ fruit were higher than those in ‘Benihoppe’. In addition, the transcript abundances of *FaAAT* and *FaAAT1* were higher in ‘Xiaobai’ than those in ‘Benihoppe’ in the white fruit, turning fruit and red fruit stages (Figure 4B). *FcAAT1* plays an important role in strawberry flavor, and this gene is positively correlated with ester synthesis [46]. *FaAAT2* is involved in the synthesis of fruit volatile substances in strawberries, and the expression pattern of this gene during receptacle growth and ripening is consistent with the production of esters during the ripening of strawberry fruit [47]. In this study, we found that the expression levels of *FaAAT* and *FaAAT1* were higher than *FaAAT2*, suggesting that *FaAAT* and *FaAAT1* play an important role in ester synthesis in ‘Benihoppe’ and ‘Xiaobai’ (Figure 4B).

Eugenol is a type of phenylpropene that plays different roles in different organs of plants. It can induce insect pollination and defend against animals and microorganisms [48]. Aromatic compounds, such as phenylpropenes, are produced by fruits [49]. In strawberries, eugenol production in the ripening fruit has been reported [50]. In this study, the content of eugenol in the red fruit stage of ‘Benihoppe’ was higher than that in ‘Xiaobai’ (Tables S2 and S3). *FaEGS1a* and *FaEGS2* are the key genes for the synthesis of eugenol and isoeugenol, respectively [36]. A recent study showed that eugenol synthase 1 (EGS1) and eugenol synthase 2 (EGS2) have the same catalytic activity, but they have opposite expression patterns [51]. The expression level of *FaEGS1a* is higher in the green fruit stage and significantly decreased in the white fruit and red fruit stages, whereas the expression level of *FaEGS2* is lower in the green fruit and white fruit stages and higher in the red fruit stage [36], which is consistent with our results. Taken together, the higher expression of *FaEGS1a* in ‘Benihoppe’ than that in ‘Xiaobai’ may result in the increased content of eugenol in ‘Benihoppe’ compared with ‘Xiaobai’. In addition, the content of benzene and volatile phenol in ‘Xiaobai’ was 5.93 times higher than that in ‘Benihoppe’ and the content of anthocyanins in ‘Xiaobai’ flesh was significantly decreased compared with ‘Benihoppe’ [52]. Phenylalanine ammonia-lyase (PAL) participates in both the flavonoid biosynthetic pathway and the phenylpropanoid/benzenoid biosynthetic pathway, which might be a trade-off relationship between them.

Terpenoids play an important role in the characteristic aroma of strawberries [6]. The main terpenoids in cultivated strawberries are monoterpenoid linalool and sesquiterpenoid nerolidol [23]. The biosynthesis of nerolidol and linalool is based on isopentenyl diphosphate (IPP) and dimethylallyl diphosphate (DMAPP). *FaNES1* is an important gene for the synthesis of nerolidol and linalool [53]. In this study, the contents of linalool and nerolidol in ‘Xiaobai’ were higher than those in ‘Benihoppe’ in the red fruit stage (Tables S2 and S3). The OAVs of linalool and nerolidol in ‘Xiaobai’ were higher than those in ‘Benihoppe’ (Table 1). It has been reported that the synthesis of linalool and nerolidol may be positively correlated with the transcriptional level of the *FaNES1* gene. We found that the expression of *FaNES1* increased with fruit development. However, we found no significant difference in the expression of *FaNES1* between ‘Benihoppe’ and ‘Xiaobai’. Next, we detected the transcript abundances of other genes involved in the terpenoid pathway in the fruit of ‘Benihoppe’ and ‘Xiaobai’ at different developmental stages. Interestingly, the expression levels of *FaDXS*, *FaMCS,* and *FaHDR* in ‘Xiaobai’ were significantly increased compared with ‘Benihoppe’ in the red fruit stage, which might lead to increased linalool and nerolidol in ‘Xiaobai’.

## 4. Materials and Methods

### 4.1. Plant Materials

The strawberry materials were ‘Benihoppe’ and ‘Xiaobai’. ‘Xiaobai’ was a cultivar produced from the tissue culture of apices of stolon tips of ‘Benihoppe’, which was from the Li Jian of Beijing Aoyi Kaiyuan vegetable planting cooperative. ‘Benihoppe’ and ‘Xiaobai’ were cultivated in the greenhouse of Shenyang Agricultural University, China. The fruit was divided into four stages at 8 days (green fruit stage), 16 days (white fruit stage), 24 days (turning fruit stage), and 32 days (red fruit stage) after pollination [54]. The fruit samples of ‘Benihoppe’ and ‘Xiaobai’ from four stages were used for RT-qPCR and HS-SPME-GC-MS analysis.

### 4.2. Volatile Compounds Extraction

The extraction of volatile compounds from fruit samples was based on the methods described in [54]. We weighed 50 g of the powder, then blended this with 0.5 g of D-glucolactone (to inhibit glycosidase activity) and 1 g of PVPP (to remove polyphenols and prevent sample oxidation). To obtain a clear juice, the powder was immediately centrifuged at 8000 rpm at 4 °C for 15 min after maceration at 4 °C for 240 min.

### 4.3. HS-SPME-GC-MS Analysis

The detailed method used for the identification of volatile compounds in strawberries using HS-SPME-GC-MS described in [54] was followed.

### 4.4. Quantitative Real-Time PCR

Total RNA was extracted from the fruit at four developmental stages of ‘Benihoppe’ and ‘Xiaobai’ using a modified CTAB method as described in [55]. We tested the RNA concentration and RNA quality using an ultramicro ultraviolet spectrophotometer (NanoDrop 20,000.5 μL, 190–840 nm). The genome DNA was removed by enzymolysis. The reaction contained 2.0 μL 5 × gDNA Eraser Buffer, 1.0 μL gDNA Eraser, 1 μg RNA and RNase-free ddH_2_O to 10 μL. The reaction was conducted at 42 °C for 5 min. Total RNA (1 μg) was reverse-transcribed using a PrimeScript^TM^ RT kit (TaKaRa, Dalian, China), and the volume of the cDNA was 20 uL. The gene sequences involved in volatile biosynthetic pathways were based on the published literature [4,6]. Then, we used these sequences as baits for BLAST on the *Fragaria* × *ananassa* database (https://www.rosaceae.org/, accessed on 20 June 2021) [56,57]. The sequences from *Fragaria* × *ananassa* with the highest similarity were selected, and RT-qPCR primers were designed using the online software Primer 3.0 based on the conserved sequences. The sequences of primers used in this study are listed in Table S1. We performed quantitative real-time RT-PCR (RT-qPCR) using the QuantStudio^TM^ 6 Flex system (Applied Biosystems, Foster City, CA, USA) according to the manufacturer’s instructions. Measurements of the gene expression levels of aroma-related genes were carried out using SYBR^®^ Premix Ex Taq TM II (CWBio, Beijing, China). The reaction system was established as described in [53]. Strawberry 26S rRNA (*Fa26S*) is a housekeeping gene in strawberries and was selected as a reference gene [58,59]. Each sample was analyzed in triplicate with three biological replicates. The ^2−∆∆^Ct method was used for calculating the relative gene expression level [60].

### 4.5. Statistical Analysis

Statistical analysis was performed using IBM SPSS Statistics 19. Three replicates were used for each measurement. Based on Duncan’s test (*p* < 0.05), different letters represent significant differences between different treatments. For the nine categories of volatile compounds, a T-test was used to determine whether there was a significant difference between the two treatments (*, *p* < 0.05; **, *p* < 0.01). SigmaPlot 12.5 (Systat Software, Inc., San Jose, CA, USA) was used to visualize the data. Principal component analysis was conducted using Origin 8.0 software (MicroCal Software Inc., Northampton, MA, USA). We analyzed the results of RT-qPCR using Microsoft Excel, and the heatmap of gene expression of fruit at four developmental stages was generated using TBtools1.082 [61].

## 5. Conclusions

As shown in Figure S1, the gene expression levels of *FaHPL* and *FaADH* gradually increased in the early stages of fruit development, which was consistent with the higher contents of aldehydes and alcohols, and was responsible for the grassy aroma in the early stages of fruit development in ‘Benihoppe’ and ‘Xiaobai’. With the growth and development of strawberry fruit, the transcript levels of *FaHPL* and *FaADH* decreased and the gene expression levels of *FaAAT* and *FaAAT1* increased. Therefore, the contents of aldehydes and alcohols gradually decreased, whereas the content of esters significantly increased, which led to the strong fragrance of ‘Benihoppe’ and ‘Xiaobai’ in the red fruit stage.

In addition, the expression of *FaEGS1a* in the red fruit stage of ‘Benihoppe’ was higher than that in ‘Xiaobai’, and the content of eugenol in ‘Benihoppe’ was higher than that in ‘Xiaobai’, which may be one of the reasons for the difference in aroma between ‘Benihoppe’ and ‘Xiaobai’. Higher contents of esters, linalool, and nerolidol in the red fruit stage of ‘Xiaobai’ were related to the increased expression of genes involved in the fatty acid pathway (*FaADH, FaAAT,* and *FaAAT1*) and the terpenoid pathway (*FaDXS, FaMCS,* and *FaHDR*) in the red fruit stage of ‘Xiaobai’ compared with ‘Benihoppe’. The results provide insights into the somaclonal variations that affect the volatile compounds in strawberries and can be used for strawberry quality improvement.

## Figures and Tables

**Table 1:** Odor activity values (OAVs) of selected aroma compounds in ‘Benihoppe’ and ‘Xiaobai’.

Aroma Components	Threshold (mg·kg^−1^)	WT-Green	WT-White	WT-Turning	WT-Red	mut-Green	mut-White	mut-Turning	mut-Red
Ethyl butyrate	0.001 ^bde^	1.50	0.90	3.60	127.80	1.30	2.20	10.00	4421.60
Ethyl isovalerate	0.002 ^e^	0.00	0.00	0.00	3.70	0.00	0.00	0.55	542.15
Ethyl pentanoate	0.0015 ^c^	0.00	0.00	0.00	1.00	0.27	0.40	0.67	24.87
Ethyl hexanoate	0.0003 ^ad^	7.00	2.00	6.00	226.33	1.67	2.67	22.67	19,548.33
Ethyl Acetate	1 ^bc^	0.01	0.01	0.01	0.03	0.02	0.02	0.02	3.75
Hexyl acetate	0.002 ^ade^	0.20	0.20	4.85	34.50	0.00	0.60	7.50	52.10
Methyl butyrate	0.01 ^bc^	0.00	0.00	1.76	45.72	0.00	0.42	6.07	42.76
Methyl hexanoate	0.087 ^ad^	0.01	0.00	0.04	4.20	0.00	0.04	0.55	6.07
Hexanal	0.1 ^b^	5.50	3.55	7.41	6.23	0.92	6.82	4.62	6.94
Octanal	0.001 ^e^	1.10	1.10	1.80	4.30	0.50	1.20	1.70	10.00
Nonanal	0.001 ^e^	0.50	0.40	0.50	0.90	0.30	0.50	0.50	1.30
Linalool	0.001 ^bcd^	1.50	1.30	20.70	146.90	0.60	4.50	36.30	528.00
Nerolidol	0.1 ^b^	0.00	0.00	0.00	0.06	0.00	0.00	0.01	1.47
Hexanol	0.1 ^b^	0.94	0.08	0.13	0.37	0.12	0.19	0.15	0.10
cis-3-Hexenol	0.03 ^c^	0.53	0.03	0.03	0.04	0.19	0.11	0.05	0.04
trans-2-Hexenol	1 ^b^	0.00	0.00	0.00	0.01	0.00	0.00	0.00	0.02
1-Octanol	0.11 ^e^	0.02	0.02	0.04	0.07	0.01	0.03	0.03	0.09
Acetic acid	100 ^b^	0.00	0.00	0.00	0.00	0.00	0.00	0.00	0.00
Butanoic acid	1 ^b^	0.00	0.00	0.00	0.03	0.00	0.00	0.00	0.04
Hexanoic acid	10 ^b^	0.00	0.00	0.01	0.11	0.00	0.00	0.02	0.34
Heptylic acid	0.64 ^c^	0.00	0.00	0.00	0.00	0.00	0.00	0.00	0.01
Octanoic acid	0.91 ^c^	0.00	0.00	0.00	0.12	0.00	0.00	0.01	0.37
Benzaldehyde	0.35 ^e^	0.01	0.01	0.02	0.04	0.00	0.01	0.03	0.38
Benzyl acetate	0.75 ^e^	0.00	0.00	0.00	0.00	0.00	0.00	0.00	0.01
Methyl salicylate	0.04 ^c^	0.03	0.01	0.02	0.09	0.01	0.01	0.05	0.26
Benzyl alcohol	0.62 ^c^	0.00	0.00	0.00	0.00	0.00	0.00	0.00	0.00
trans-3-Hexenyl acetate	0.016 ^c^	0.00	0.03	0.03	0.19	0.00	0.07	0.03	0.19
trans-2-Hexenyl acetate	0.21 ^c^	0.00	0.00	0.05	0.52	0.00	0.00	0.11	0.33
Butyl butylate	0.11 ^c^	0.00	0.00	0.00	0.05	0.00	0.00	0.00	0.14
Hexyl butyrate	0.25 ^ae^	0.00	0.00	0.00	0.00	0.00	0.00	0.00	0.00

The thresholds for the 30 volatiles are from the following literature: ^a^ [29], ^b^ [7], ^c^ [2], ^d^ [8], ^e^ [14]. The contents of volatiles are shown in Supplementary Tables S2 and S3. (WT: ‘Benihoppe’; mut: ‘Xiaobai’).

## Data Availability

The data presented in this study are available on request from the corresponding author.

## Supplementary Materials

The following supporting information can be downloaded at https://www.mdpi.com/article/10.3390/plants12051109/s1, Figure S1: Overview of differential volatile compounds and possible key genes responsible for the differential volatile compounds between ‘Benihoppe’ and ‘Xiaobai’. Table S1: Primers used for gene expression analysis. Table S2: Relative contents of volatile components in four periods of ‘Benihoppe’ (WT). Table S3: Relative contents of volatile components in four periods of somaclonal mutant ‘Xiaobai’ (mut). Table S4: The proportion of nine categories of volatile compounds in four developmental stages of fruit between ‘Benihoppe’ (WT) and somaclonal mutant ‘Xiaobai’ (mut). Table S5: The ratio of volatile substances contents in ‘Benihoppe’ (WT) and somaclonal mutant ‘Xiaobai’ (mut) in four periods. Table S6: The unique volatile components in ‘Benihoppe’ (WT). Table S7: The unique volatile components in somaclonal mutant ‘Xiaobai’ (mut). Table S8: Principal component load matrix of volatile compounds.
