# Supplementary material for: Comparative Characterization of Fruit Volatiles and Volatile-Related Genes Expression of ‘Benihoppe’ Strawberry and Its Somaclonal Mutant
Source: Plants (Basel). 2023 Mar 1;12(5):1109. doi: 10.3390/plants12051109 (PMC10005569; doi:10.3390/plants12051109)
Supplement: Supplementary file 1 [file plants-12-01109-s001.zip › plants-2240338-supplementary.pdf]

Supplementary materials

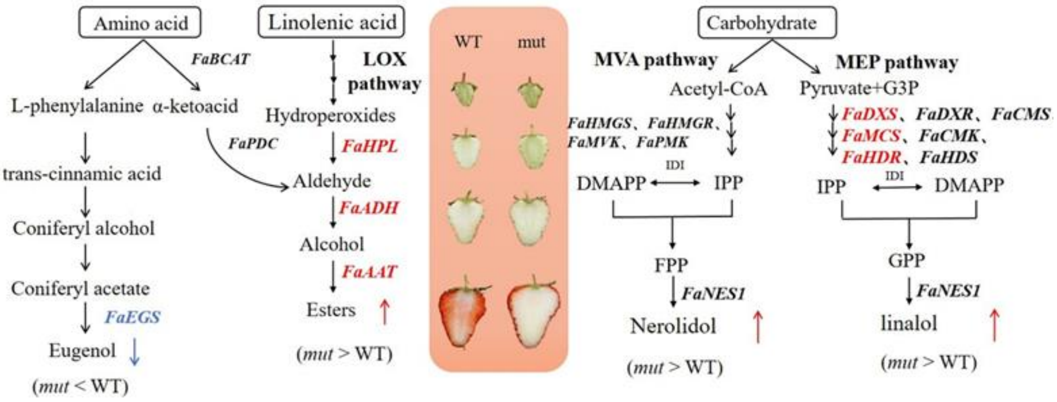

Figure S1. Overview of differential volatile compounds and possible key genes responsible for the differential volatile compounds between 'Benihoppe' and 'Xiaobai'.The blue arrow represents decreased volatile compounds in the red fruit stage of 'Xiaobai' compared with 'Benihoppe' and the blue font represents the gene may result in decreased volatile compounds in the red fruit stage of 'Xiaobai'. The red arrow represents increased volatile compounds in the red fruit stage of 'Xiaobai' compared with 'Benihoppe' and the red font represents the genes may result in increased volatile compounds in the red fruit stage of 'Xiaobai'.

Table S1. Primers used for gene expression analysis.

Primer Names	Primer Sequence (5'-3')
FaACX1-qPCR-F	CAAACAAGGTTCACTCGCCA
FaACX1-qPCR-R	CGCCTCCTCGTAAAGTTTCG
FaACX2-qPCR-F	ACTGCTCGATGGGAAAGTGA
FaACX2-qPCR-R	CAGCTCGAGAACTTGCATCC
FaACX4-qPCR-F	GCAACAAAGGTGGAAGGAGG
FaACX4-qPCR-R	TCCATTTTGAACCATGCGCA
FaFAD2-qPCR-F	GTCCCTTGTTGGTGGTGAAC
FaFAD2-qPCR-R	AAGGCTCCTCTCAACCAGTC
FaFAE4-LIKE-qPCR-F	AATGGGGTGTAGTGCTGGAG
FaFAE4-LIKE-qPCR-R	CGACGCCTATCCAATGACTT
FaFAH1-qPCR-F	GCCCCACTTTTATTGCCACA
FaFAH1-qPCR-R	CGACTCACCCTCACCTCTTT
FaPXG-qPCR-F	CTTTGGCAACATCAGCAAAA
FaPXG-qPCR-R	CAAGAGCTCGACATCCAACA
FaPXG4-qPCR-F	TTTCATCAAGCATGCACACA
FaPXG4-qPCR-R	*GCTCCCATCGTAGACAGCTC*
FaCYP1-qPCR-F	*TAGGGTAGTGGGAGTCGACA*
FaCYP1-qPCR-R	*GCTCCACTTGTTGACATGCA*
FaEH2-LIKE-qPCR-F	*GTGCTTTTCCTGCATGGATT*
FaEH2-LIKE-qPCR-R	*CCAAAAGGCCAACAACATCT*
FvLOX1-qPCR-F	*GGGAGTCCAGACAAAGGTGA*
FvLOX1-qPCR-R	*TTCAAGTTTGCGGCTCTTCC*
FvLOX2-qPCR-F	*AGAGTGTGCTGACTATCCCG*
FvLOX2-qPCR-R	*GGAATCCGGTGAGCTCGATA*
FaLOX3-qPCR-F	*TTGGGAGGCTAAAGGGAGTG*
FaLOX3-qPCR-R	*AAATTCGTCATCTCGCAGCC*
FaLOX5-qPCR-F	*GAAGAGCTCCGCAATGAAGG*
FaLOX5-qPCR-R	*GGCCACATCAGGGTTAGTCT*
FaLOX6-qPCR-F	*GAACTAGCTAGGCCGGTCAT*

FaLOX6-qPCR-R	*GCAAGGCTTTCAACTTCCCA*
FaHPL-qPCR-F	*AACGCATTTGGTGGTTTCTC*
FaHPL-qPCR-R	*GTTGAGCCGGAGTGTTTCAT*
FaADH-qPCR-F	*GAACCCCAAAGACCACAAGA*
FaADH-qPCR-R	*AAGAACAGCAACACCCCAAC*
FaAAT1-qPCR-F	*GCAATCGGTGTCTTCGTCTC*
FaAAT1-qPCR-R	*ATCTCTCGGTGGGAAAAGCA*
FaAAT2-qPCR-F	*GGCTCTGATGGCATTCTTGG*
FaAAT2-qPCR-R	*GGACCGTTGCACCATAGTTC*
FaAAT-qPCR-F	*TGCTTTTCCCACCGAGAGAT*
FaAAT-qPCR-R	*AAACCAGTGACGGCATGAAC*
FaPDC-qPCR-F	*CACCTCCACCGTTCAAAACT*
FaPDC-qPCR-R	*GAGGTGGTCAAGAAGGGTCA*
FaPDC1-qPCR-F	*TGCCTTTTGTGCTGAGATTG*
FaPDC1-qPCR-R	*TTGCTAGGCCTACGAGGAAA*
FaBCAT2-qPCR-F	*CAACGGCCGGGACTATACTA*
FaBCAT2-qPCR-R	*AACAGTTTGGGCACCTGTTC*
FaPAL1-qPCR-F	*GCTGAGCAACACAACCAAGA*
FaPAL1-qPCR-R	*GTCCTCCTGGCCACTTTACT*
FaPAL2-qPCR-F	*AGTCTCACACTCTGCCTCAC*
FAPAL2-qPCR-R	*AGGCAAGCAGGGAGTAATGT*
FaEGS1a-qPCR-F	*GGTTGAGCCGGCTAAATCTG*
FaEGS1a-qPCR-R	*TGTAGGTGCCAATGTCGTCT*
FaEGS2-qPCR-F	*GCAGAGAAGTTGAGGCATCG*
FaEGS2-qPCR-R	*TTAAGGTTCTCGGGTCGTCC*
FaDXS-qPCR-F	*CACAAAGACCACCCACTCCT*
FaDXS-qPCR-R	*ACAACACCAAGGCTTGATCC*
FaDXR-qPCR-F	*AGCAAATGAAAAGGCTGTGG*
FaDXR-qPCR-R	*CGTGCCCACAAATCATAGTG*
FaMCS-qPCR-F	*CCCAGAACCAAACCCCTACT*
FaMCS-qPCR-R	*CCACCACACAATGAAGCAAC*
FaCMS-qPCR-F	*GCAGCTGTACTAGGCGTTCC*
FaCMS-qPCR-R	*ACAGGATGTGGAAGGTGCTC*
FaCMK-qPCR-F	*AAGTGCCTACAGGAGCAGGA*
FaCMK-qPCR-R	*CAAAGGGTACAGGTGGAGGA*
FaHDS-qPCR-F	*GATAGACGCGCACAGTTTGA*
FaHDS-qPCR-R	*CTCGCAAATTCAAATGCAGA*
FaHDR-qPCR-F	*GGTGTTCCGGAAGAATCTCA*
FaHDR-qPCR-R	*TCTGGCCTCGTAAGCAATCT*
FaHMG-CoA-qPCR-F	*TGGTTCAACTGCAACCATGT*
FaHMG-CoA-qPCR-R	*CCTCCATACCTGTGCTCCAT*
FaHMGR2-qPCR-R	*ATAGCCCACTGGCATTTCAC*
FaHMGR2-qPCR-R	*ATAGCCCACTGGCATTTCAC*
FaMVK-qPCR-F	*GGAAGTCAAAGCCAGAGCAC*
FaMVK-qPCR-R	*GTTGATGGGTTGGGAACATC*
FaPMK-qPCR-F	*CCGAGGAATCAAATGGAGAA*
FaPMK-qPCR-R	*CAAAACCACTGCCCACTTTT*
FaCCD1-qPCR-F	*ATGCTTGGGAGGAAGAGGAC*
FaCCD1-qPCR-R	*TCTGTGTTGCTAGACCGGTT*
FaCCD4-qPCR-F	*GCAGGATGTACGGTGAGGAT*
FaCCD4-qPCR-R	*GATGTCCAGCTCAGACGACA*
FaNES1-qPCR-F	*AGTATTGTGTCTGCCCGTGA*
FaNES1-qPCR-R	*CCTTCTGTACCTAGCTGCGA*

Table S2. Relative contents of volatile components in four developmental stages of 'Benihoppe' (WT).

Aroma components	RI	Classification	WT -Green	WT -White	WT -Turn	WT -Red
Methoxypropanol	1131	*Alcohols*	0.00390±0.00155ab	0.00020±0.00029c	—	—
Pentyl alcohol	1256	*Alcohols*	0.00200±0.00047c	0.00310±0.00037c	0.00830±0.00125a	0.00890±0.00012a
2-Heptanol	1327	*Alcohols*	0.00680±0.00066c	0.00380±0.00073cd	0.00620±0.00026cd	0.01910±0.00050b
cis-2-Pentenol	1309	*Alcohols*	0.00810±0.00041a	0.00350±0.00016c	0.00500±0.00042b	0.00390±0.00004c
Hexanol	1356	*Alcohols*	0.09440±0.00200a	0.00800±0.00126g	0.01340±0.00104de	0.03720±0.00168b
cis-3-Hexenol	1371	*Alcohols*	0.01590±0.00034a	0.00100±0.00008e	0.00100±0.00007e	0.00110±0.00011e
trans-2-Hexenol	1324	*Alcohols*	0.00260±0.00038b	0.00200±0.00033b	0.00290±0.00044b	0.00670±0.00016b
cis-2-Hexenol	1398	*Alcohols*	0.05490±0.00113a	0.00550±0.00246de	0.00790±0.00070cd	0.01910±0.00075b
1-Heptanol	1460	*Alcohols*	0.00160±0.00008b	0.00070±0.0001e	0.00170±0.00003b	0.00280±0.00005a
2-Ethyl-1-hexanol	1489	*Alcohols*	0.01260±0.00032a	0.01140±0.0007a	0.00910±0.00038b	0.00880±0.00032b
3-Ethyl-4-methylpentanol	1500	*Alcohols*	0.00030±0.00002b	0.00010±0.00001b	0.00030±0.00003b	0.00030±0.00003b
1-Octanol	1557	*Alcohols*	0.00210±0.00003e	0.00220±0.00023de	0.00450±0.00026c	0.00790±0.00029bc
trans-2-Octenol	1616	*Alcohols*	0.00100±0.00006e	0.00120±0.00008de	0.00220±0.00005c	0.00230±0.00023bc
Nonanol	1654	*Alcohols*	0.00070±0.00005b	0.00080±0.00014b	0.00070±0.00006b	0.00110±0.00013a
1-Dodecanol	1404	*Alcohols*	0.00020±0.00004c	0.00010±0.00004c	0.00060±0.00037ab	0.00030±0.00008bc
2-methyl-5-Hepten-1-ol	1730	*Alcohols*	—	—	—	0.00050±0.00002b
Acetic acid	1449	*Acid*	0.00160±0.00029b	0.00080±0.00011b	0.00130±0.00027b	0.00360±0.00039b
Isobutyric acid	1557	*Acid*	0.00030±0.00003cd	0.00020±0.00002de	0.00030±0.00001cd	0.00290±0.00019b
Butanoic acid	1574	*Acid*	0.00020±0.00008c	0.00010±0.00001c	0.00070±0.00008c	0.02650±0.00249b
Methylethylacetic acid	1658	*Acid*	0.00060±0.00041c	0.00010±0.00003c	0.00380±0.00006c	0.08650±0.00634a
Hexanoic acid	1762	*Acid*	0.01700±0.00897d	0.00290±0.00076d	0.05340±0.00323cd	1.10860±0.07603b
trans-3-Hexenoic Acid	1909	*Acid*	—	—	—	0.00050±0.00008b
Heptylic acid	1947	*Acid*	0.00020±0.00002c	0.00010±0.00002c	0.00030±0.00001c	0.00200±0.00022b
trans-2-Hexenoic acid	1933	*Acid*	0.00180±0.00020bc	0.00070±0.00011c	0.00140±0.00024c	0.00290±0.00024b
Octanoic acid	2052	*Acid*	0.00210±0.00112c	0.00050±0.00011c	0.00320±0.00019c	0.11210±0.01241b
Nonanoic acid	2155	*Acid*	0.00070±0.00005b	0.00040±0.00006b	0.00030±0.00005b	0.00100±0.00014b
trans-2-Octenoic acid	2182	*Acid*	—	—	—	—
Ethyl butyrate	1039	*Ethyl esters*	0.00150±0.00047b	0.00090±0.00042b	0.00360±0.00060b	0.12780±0.00642b
Ethyl 2-methylbutyrate	1062	*Ethyl esters*	—	—	—	0.00090±0.00007b
Ethyl isovalerate	1093	*Ethyl esters*	—	—	—	0.00740±0.00035b
Ethyl pentanoate	1140	*Ethyl esters*	—	—	—	0.00150±0.00002b

Ethyl hexanoate	899	*Ethyl esters*	0.00210±0.00138b	0.00060±0.00015b	0.00180±0.00052b	0.06790±0.00463b
Ethyl 2-hexenoate	1343	*Ethyl esters*	—	—	—	—
Ethyl 3-hydroxyhexanoate	1677	*Ethyl esters*	—	—	—	—
Methyl acetate	848	*acetate esters*	0.00650±0.00026f	0.00720±0.00079f	0.14610±0.01222d	0.33920±0.00789a
Ethyl Acetate	764	*acetate esters*	0.00790±0.00076b	0.00760±0.0007b	0.01330±0.00139b	0.02590±0.00071b
Isopentyl acetate	1136	*acetate esters*	—	—	0.00820±0.00080c	0.03110±0.00203b
isopentenyl acetate	1249	*acetate esters*	—	—	—	0.00350±0.00007b
2-Heptyl acetate	1380	*acetate esters*	—	—	—	0.00110±0.00014b
Hexyl acetate	1299	*acetate esters*	0.00040±0.00012d	0.00040±0.00006d	0.00970±0.00227cd	0.06900±0.00463b
trans-3-Hexenyl acetate	1306	*acetate esters*	—	0.00040±0.00004c	0.00050±0.00007c	0.00300±0.00013a
trans-2-Hexenyl acetate	1337	*acetate esters*	0.00030±0.00007e	0.00030±0.00005e	0.01040±0.00173d	0.10870±0.00485a
Methyl butyrate	945	*other esters*	—	—	0.01760±0.00229d	0.45720±0.01131a
Methyl isopentanoate	1019	*other esters*	—	—	0.00180±0.00028d	0.03310±0.00151b
Propyl isovalerate	1167	*other esters*	—	—	—	—
Methyl hexanoate	1197	*other esters*	0.00120±0.00070d	0.00040±0.00004d	0.00330±0.00109d	0.36510±0.01481b
Butyl butylate	1240	*other esters*	—	—	—	0.00500±0.00095b
Isoamyl butylate	1263	*other esters*	—	—	—	0.00410±0.00040b
Methyl 2-hexenoate	1272	*other esters*	0.00090±0.00013cd	0.00080±0.0001cd	0.00150±0.00016c	0.00490±0.00025b
Hexyl butyrate	1417	*other esters*	0.00050±0.00012b	0.00030±0.00004b	0.00060±0.00007b	0.00050±0.00012b
trans-2-Hexenyl Butyrate	1476	*other esters*	—	—	0.00030±0.00002c	0.00120±0.00008b
Octyl isovalerate	1654	*other esters*	—	—	—	—
p-Xylene	870	*Benzene and volatile phenols*	0.00760±0.00042cd	0.00420±0.00080de	0.01230±0.00289b	0.01870±0.00178a
Styrene	1264	*Benzene and volatile phenols*	0.00080±0.00005bc	0.00050±0.00009c	0.00080±0.00007bc	0.00180±0.00098b
Benzaldehyde	1523	*Benzene and volatile phenols*	0.00310±0.00066c	0.00180±0.00018c	0.00580±0.00074c	0.01390±0.00065b
Clorius	1590	*Benzene and volatile phenols*	—	—	0.00040±0.00001c	0.00070±0.00002b
Benzeneacetaldehyde	1574	*Benzene and volatile phenols*	0.00340±0.00028b	0.00040±0.00005c	—	—
Acetophenon	1652	*Benzene and volatile phenols*	0.00080±0.00005b	0.00060±0.00006bc	0.00030±0.00002d	0.00050±0.00004cd
Ethyl benzoate	1671	*Benzene and volatile phenols*	—	—	—	—
Benzyl acetate	1727	*Benzene and volatile phenols*	—	—	0.00030±0.00001cd	0.00140±0.00005b
Naphthalene	1744	*Benzene and volatile phenols*	0.00630±0.00020b	0.00370±0.00039cde	0.00320±0.00031de	0.00540±0.00062bc
Methyl salicylate	1675	*Benzene and volatile phenols*	0.00130±0.00022d	0.00030±0.00005e	0.00060±0.00001e	0.00360±0.00019b

2-Methylnaphthalene	1839	*Benzene and volatile phenols*	0.00200±0.00007b	0.00110±0.00011cd	0.00100±0.0001cd	0.00160±0.00025bc
Benzyl alcohol	1883	*Benzene and volatile phenols*	0.00180±0.00016a	0.00030±0.00002d	0.00100±0.00008bc	0.00190±0.00014a
Phenylethyl Alcohol	1920	*Benzene and volatile phenols*	0.00130±0.00013a	0.00020±0.00002d	0.00040±0.00004cd	0.00100±0.00011b
Benzenol	2012	*Benzene and volatile phenols*	0.00030±0.00003c	0.00020±0.00001d	0.00030±0.00001c	0.00040±0.00001b
Ethyl cinnamate	2145	*Benzene and volatile phenols*	0.00070±0.00025b	0.00020±0.00006b	0.00030±0.00008b	0.00300±0.00026b
Cinnamyl acetate	2192	*Benzene and volatile phenols*	—	—	—	—
Eugenol	2160	*Benzene and volatile phenols*	—	—	—	0.00050±0.00003a
Pentanal	982	*aldehydes and ketone*	0.00410±0.00015e	0.00420±0.00050e	0.01530±0.00173c	0.03050±0.00085b
Ethyl vinyl ketone	1410	*aldehydes and ketone*	0.00480±0.00021e	0.01060±0.00122e	0.08180±0.00825a	0.04820±0.00186c
Hexanal	1041	*aldehydes and ketone*	0.55010±0.09598bc	0.35450±0.05897d	0.74080±0.03536a	0.62290±0.03214ab
trans-2-Pentenal	1173	*aldehydes and ketone*	0.01130±0.00252de	0.00920±0.00028e	0.02320±0.00174b	0.01710±0.00184c
3-Hexenal	853	*aldehydes and ketone*	0.02410±0.00301bc	0.01320±0.00240ef	0.02810±0.00295ab	0.01670±0.00214de
Isobutyl ketone	1207	*aldehydes and ketone*	0.00590±0.00020ab	0.00470±0.00072bc	0.00450±0.00082bc	0.00640±0.00083a
Heptanal	1187	*aldehydes and ketone*	0.00360±0.00029cd	0.00250±0.00039d	0.00520±0.00026c	0.01430±0.00151b
trans-2-Hexenal	1329	*aldehydes and ketone*	0.03680±0.00097a	0.01600±0.00230de	0.02810±0.00218b	0.01690±0.00081d
2-Hexenal	1220	*aldehydes and ketone*	1.51500±0.03934a	0.74210±0.088970e	1.21590±0.05420b	0.89540±0.03303cd
3-Octanone	1281	*aldehydes and ketone*	0.00070±0.00004cd	0.00060±0.00011cd	0.00200±0.00055c	0.00750±0.00027b
Octanal	1280	*aldehydes and ketone*	0.00110±0.00014c	0.00110±0.00014c	0.00180±0.00009c	0.00430±0.00021b
1-Octene-3-one	1438	*aldehydes and ketone*	0.00080±0.00001d	0.00120±0.00024d	0.00550±0.00077b	0.00400±0.00018c
cis-2-Heptenal	1323	*aldehydes and ketone*	0.00380±0.00016d	0.00500±0.00066d	0.01360±0.00111b	0.01190±0.00018bc
Sulcatone	1333	*aldehydes and ketone*	0.00160±0.00006e	0.00140±0.00010e	0.00370±0.00048c	0.00530±0.00013b
Nonanal	1386	*aldehydes and ketone*	0.00050±0.00006c	0.00040±0.00008cd	0.00050±0.00008c	0.00090±0.00007b
Sorbaldehyde	1411	*aldehydes and ketone*	0.01470±0.00064a	0.00690±0.00088de	0.01070±0.00026c	0.00740±0.00007d
trans-2-Octenal	1428	*aldehydes and ketone*	0.00100±0.00013d	0.00160±0.00022d	0.00910±0.00045b	0.00920±0.00012b
trans-2-trans-4-Heptadienal	1494	*aldehydes and ketone*	0.01080±0.00018c	0.00620±0.00053f	0.01330±0.00121b	0.00760±0.00022e
Decanal	1504	*aldehydes and ketone*	0.00050±0.00010c	0.00040±0.00013c	0.00030±0.00008c	0.00100±0.00025b

Camphor	1516	*aldehydes and ketone*	0.00120±0.00002a	0.00040±0.00003f	0.00090±0.00003c	0.00060±0.00002e
trans-2-Nonenal	1679	*aldehydes and ketone*	0.00050±0.00003de	0.00040±0.00007de	0.00120±0.00020cd	0.00240±0.00006b
Cucumber aldehyde	1589	*aldehydes and ketone*	0.00120±0.00009d	0.00080±0.00007de	0.00270±0.00031c	0.00250±0.00012c
trans-2-Decenal	1206	*aldehydes and ketone*	0.00040±0.00005cd	0.00030±0.00010cd	0.00040±0.00007cd	0.00080±0.00006b
trans, trans-2,4-Nonadienal	1703	*aldehydes and ketone*	0.00060±0.00002cd	0.00050±0.00003de	0.00150±0.00055b	0.00160±0.00012b
1-Pentene-3-ol	1162	*Isoprenoids*	0.01170±0.00155a	0.00690±0.00066cd	0.01010±0.00115ab	0.00840±0.00039bc
β-Myrcene	1170	*Isoprenoids*	—	—	—	—
D-Limonene	1203	*Isoprenoids*	—	—	0.00050±0.00007c	0.00250±0.00020b
β-Ocimene	1246	*Isoprenoids*	—	—	0.00030±0.00004c	0.00200±0.00014b
Dihydrolinalool	1255	*Isoprenoids*	0.00250±0.00020b	0.00160±0.00024cd	0.00080±0.00027e	0.00190±0.00013bc
cis-Linalool oxide	1441	*Isoprenoids*	0.00010±0.00005f	0.00010±0.00000f	0.00090±0.00007b	0.00070±0.00004c
Morillol	1452	*Isoprenoids*	0.01440±0.00021e	0.01940±0.00204d	0.03260±0.00139c	0.03700±0.00132b
trans-Linalool oxide	1464	*Isoprenoids*	0.00020±0.00003e	0.00020±0.00001e	0.00210±0.00029b	0.00180±0.00009bc
Linalol	1527	*Isoprenoids*	0.00150±0.00009e	0.00130±0.00005e	0.02070±0.00154d	0.14690±0.00638b
Levomenthol	1651	*Isoprenoids*	0.00120±0.00016b	0.00080±0.00006cd	0.00070±0.00010d	0.00110±0.00013bc
Farnesene	1749	*Isoprenoids*	0.00040±0.00009b	—	—	0.00050±0.00002b
α-Terpineol	1697	*Isoprenoids*	0.00030±0.00004c	0.00020±0.00001c	0.00080±0.00031c	0.00330±0.00046b
Bisabolene	1792	*Isoprenoids*	—	—	—	—
Nerylacetone	1865	*Isoprenoids*	—	0.00010±0.00001bc	0.00040±0.00003bc	—
Bisabolol	2207	*Isoprenoids*	—	—	—	—
β-Damascenone	1808	*Isoprenoids*	0.00510±0.00071a	0.00410±0.00014ab	0.00390±0.00401ab	0.00370±0.00061ab
Nerolidol	1961	*Isoprenoids*	—	—	—	0.00620±0.00014b
1-p-Menthen-9-al	1212	*Isoprenoids*	0.00040±0.00002de	0.00010±0.00004e	0.00060±0.00021d	0.00190±0.0002b
2-Pentylfurane	1231	*furan*	0.00550±0.00011bc	0.00410±0.00053bc	0.00890±0.00552b	0.01070±0.00095b
Furfural	1472	*furan*	0.00200±0.00003a	0.00170±0.00016b	0.0002±0.00007e	0.0002±0.00002e

Note: different letters represent comparison of the same volatile components in four periods of 'Benihoppe' (WT, Table S2) and somaclonal mutant 'Xiaobai' (*mut*, Table S3)

Table S3. Relative contents of volatile components in four developmental stages of somaclonal mutant 'Xiaobai'(*mut*).

Aroma components	RI	Classification	*mut* - Green	*mut* - White	*mut* - Turn	*mut* - Red
Methoxypropanol	1131	*Alcohols*	0.00490±0.00036a	—	0.00260±0.00045b	0.00330±0.00029b
Pentyl alcohol	1256	*Alcohols*	0.00060±0.00003d	0.00550±0.00057b	0.00600±0.00008b	—
2-Heptanol	1327	*Alcohols*	0.00310±0.00059d	0.00680±0.00116c	0.00640±0.0011cd	0.06240±0.00296a
cis-2-Pentenol	1309	*Alcohols*	0.00260±0.00004d	0.00510±0.00018b	0.00350±0.00017c	0.00370±0.00028c
Hexanol	1356	*Alcohols*	0.01190±0.00032ef	0.01880±0.00038c	0.01480±0.00075d	0.00950±0.00032fg
cis-3-Hexenol	1371	*Alcohols*	0.00580±0.00026b	0.00320±0.00002c	0.00160±0.00006d	0.00110±0.00003e
trans-2-Hexenol	1324	*Alcohols*	0.00180±0.00016b	0.00200±0.00022b	0.00210±0.00048b	0.01840±0.00619a
cis-2-Hexenol	1398	*Alcohols*	0.00900±0.00039c	0.01760±0.00031b	0.01660±0.00065b	0.00370±0.00009e
1-Heptanol	1460	*Alcohols*	0.00050±0.00004f	0.00110±0.00004d	0.00130±0.00013c	0.00290±0.00019a

2-Ethyl-1-hexanol	1489	*Alcohols*	0.01000±0.00015b	0.01210±0.00037a	0.00890±0.00060b	0.01280±0.00103a
3-Ethyl-4-methylpentanol	1500	*Alcohols*	0.00010±0.00001b	0.00010±0.00001b	0.00020±0.00003b	0.00090±0.00026a
1-Octanol	1557	*Alcohols*	0.00070±0.00002f	0.00310±0.00016a	0.00330±0.00044d	0.00960±0.00042b
trans-2-Octenol	1616	*Alcohols*	0.00020±0.00002f	0.00320±0.00010a	0.00150±0.00019d	0.00260±0.00011b
Nonanol	1654	*Alcohols*	0.00030±0.00007c	0.00110±0.00010a	0.00060±0.00011b	0.00130±0.00005a
1-Dodecanol	1404	*Alcohols*	0.00030±0.00013bc	0.00020±0.00007c	0.00020±0.00007c	0.00080±0.00006a
2-methyl-5-Hepten-1-ol	1730	*Alcohols*	—	0.00020±0.00037bc	0.00010±0.00001c	0.00160±0.00001a
Acetic acid	1449	*Acid*	0.00190±0.00025b	0.00100±0.00014b	0.00120±0.00037b	0.06710±0.00715a
Isobutyric acid	1557	*Acid*	0.00020±0.00006cde	—	0.00050±0.00008c	0.00630±0.00021a
Butanoic acid	1574	*Acid*	0.00010±0.00005c	0.00010±0.00002c	0.00110±0.00035c	0.04120±0.00155a
Methylethylacetic acid	1658	*Acid*	0.00040±0.00029c	0.00020±0.00002c	0.00500±0.00130c	0.06850±0.00123b
Hexanoic acid	1762	*Acid*	0.00870±0.00622d	0.01290±0.00088d	0.19810±0.05991c	3.44940±0.17479a
trans-3-Hexenoic Acid	1909	*Acid*	—	—	0.00020±0.00002b	0.00290±0.00079a
Heptylic acid	1947	*Acid*	0.00010±0.00003c	—	0.00050±0.00011c	0.00650±0.00159a
trans-2-Hexenoic acid	1933	*Acid*	0.00050±0.00003c	0.00160±0.0001bc	0.00150±0.00042bc	0.00500±0.00139a
Octanoic acid	2052	*Acid*	0.00080±0.00054c	0.00110±0.00009c	0.00710±0.00168c	0.34080±0.07633a
Nonanoic acid	2155	*Acid*	0.00060±0.00014b	0.00040±0.00011b	0.00050±0.00012b	0.00280±0.00109a
trans-2-Octenoic acid	2182	*Acid*	—	—	0.00030±0.00015b	0.00110±0.00014a
Ethyl butyrate	1039	*Ethyl esters*	0.00130±0.00013b	0.00220±0.00038b	0.01000±0.00171b	4.42160±0.25362a
Ethyl 2-methylbutyrate	1062	*Ethyl esters*	—	—	—	0.13210±0.01056a
Ethyl isovalerate	1093	*Ethyl esters*	—	—	0.00110±0.00025b	1.08430±0.08662a
Ethyl pentanoate	1140	*Ethyl esters*	0.00040±0.00003b	0.00060±0.00003b	0.00100±0.00012b	0.03730±0.00254a
Ethyl hexanoate	899	*Ethyl esters*	0.00050±0.00014b	0.00080±0.00003b	0.00680±0.00190b	5.86450±0.43765a
Ethyl 2-hexenoate	1343	*Ethyl esters*	—	—	—	0.08150±0.00574a
Ethyl 3-hydroxyhexanoate	1677	*Ethyl esters*	—	—	—	0.00420±0.00060a
Methyl acetate	848	*acetate esters*	—	0.05670±0.00121e	0.16890±0.01664c	0.24010±0.00803b
Ethyl Acetate	764	*acetate esters*	0.01590±0.00057b	0.01530±0.00075b	0.02000±0.00218b	3.74560±0.16729a
Isopentyl acetate	1136	*acetate esters*	—	—	0.00610±0.00103c	0.06060±0.00428a
isopentenyl acetate	1249	*acetate esters*	—	—	0.00020±0.00002c	0.00660±0.00037a
2-Heptyl acetate	1380	*acetate esters*	—	—	—	0.01140±0.00119a
Hexyl acetate	1299	*acetate esters*	—	0.00120±0.00013d	0.01500±0.00450c	0.10420±0.01036a
trans-3-Hexenyl acetate	1306	*acetate esters*	—	0.00110±0.00008b	0.00050±0.00012c	0.00310±0.00066a
trans-2-Hexenyl acetate	1337	*acetate esters*	—	0.00070±0.00002e	0.02250±0.00542c	0.07020±0.00486b
Methyl butyrate	945	*other esters*	—	0.00420±0.00028d	0.06070±0.00970c	0.42760±0.02008b
Methyl isopentanoate	1019	*other esters*	—	—	0.01420±0.00302c	0.07080±0.00488a
Propyl isovalerate	1167	*other esters*	—	—	—	0.00520±0.00042a
Methyl hexanoate	1197	*other esters*	0.00040±0.00022d	0.00380±0.00008d	0.04810±0.01455c	0.52780±0.03324a
Butyl butylate	1240	*other esters*	—	—	—	0.01570±0.00172a
Isoamyl butylate	1263	*other esters*	—	—	0.00020±0.00005c	0.00710±0.00089a
Methyl 2-hexenoate	1272	*other esters*	0.00040±0.00001d	0.00080±0.00016cd	0.00150±0.00022c	0.01000±0.00076a
Hexyl butyrate	1417	*other esters*	0.00020±0.00008b	0.00050±0.00009b	0.00040±0.00012b	0.00110±0.00034a
trans-2-Hexenyl Butyrate	1476	*other esters*	—	—	—	0.00160±0.00026a
Octyl isovalerate	1654	*other esters*	—	—	0.00007±0.00004b	0.00010±0.00006a
p-Xylene	870	*Benzene and volatile phenols*	0.00170±0.00007e	0.00910±0.00049bc	0.00810±0.00235bcd	0.01880±0.00208a
Styrene	1264	*Benzene and volatile phenols*	0.00030±0.00003c	0.00110±0.00006bc	0.00100±0.00013bc	0.00710±0.00068a
Benzaldehyde	1523	*Benzene and volatile phenols*	0.00150±0.00018c	0.00350±0.00021c	0.01130±0.00080b	0.13330±0.00444a
Clorius	1590	*Benzene and volatile phenols*	—	—	0.00030±0.00003d	0.00130±0.00006a
Benzeneacetaldehyde	1574	*Benzene and volatile phenols*	0.00060±0.00002c	0.00050±0.00004c	0.00040±0.00003c	0.00440±0.00028a
Acetophenon	1652	*Benzene and volatile phenols*	0.00050±0.00003cd	0.00030±0.00002d	0.00060±0.00005bcd	0.00510±0.00027a

Ethyl benzoate	1671	*Benzene and volatile phenols*	0.00010±0.00002b	—	—	0.01450±0.00057a
Benzyl acetate	1727	*Benzene and volatile phenols*	0.00010±0.00007e	0.00010±0.00002de	0.00040±0.00003c	0.00630±0.00024a
Naphthalene	1744	*Benzene and volatile phenols*	0.00220±0.00008e	0.00420±0.00052cd	0.00390±0.00057cde	0.01320±0.00160a
Methyl salicylate	1675	*Benzene and volatile phenols*	0.00030±0.00007e	0.00050±0.00001e	0.00210±0.00025c	0.01020±0.00052a
2-Methylnaphthalene	1839	*Benzene and volatile phenols*	0.00080±0.00004d	0.00120±0.00012cd	0.00110±0.00013cd	0.00380±0.00052a
Benzyl alcohol	1883	*Benzene and volatile phenols*	0.00090±0.00025bc	0.00080±0.00002cd	0.00140±0.00052ab	0.00130±0.00018abc
Phenylethyl Alcohol	1920	*Benzene and volatile phenols*	0.00060±0.00015c	0.00030±0.00001cd	0.00060±0.00021c	0.00050±0.00009cd
Benzenol	2012	*Benzene and volatile phenols*	0.00010±0.00001e	0.00030±0.00002cd	0.00030±0.00006c	0.00080±0.00005a
Ethyl cinnamate	2145	*Benzene and volatile phenols*	0.00020±0.00007b	0.00020±0.00003b	0.00100±0.00017b	0.10050±0.01574a
Cinnamyl acetate	2192	*Benzene and volatile phenols*	0.00020±0.00006b	—	—	0.00080±0.00027a
Eugenol	2160	*Benzene and volatile phenols*	—	—	0.00030±0.00007b	0.00010±0.00005c
Pentanal	982	*aldehydes and ketone*	0.00130±0.00002e	0.00910±0.00044d	0.01510±0.00216c	0.03470±0.00156a
Ethyl vinyl ketone	1410	*aldehydes and ketone*	0.00130±0.00004e	0.02130±0.00044d	0.05790±0.00531b	0.05350±0.00377bc
Hexanal	1041	*aldehydes and ketone*	0.09210±0.00119e	0.68160±0.01742ab	0.46200±0.08023cd	0.69380±0.04251a
trans-2-Pentenal	1173	*aldehydes and ketone*	0.00530±0.00018f	0.01340±0.00090d	0.01960±0.00102c	0.02890±0.00109a
3-Hexenal	853	*aldehydes and ketone*	0.00320±0.00032g	0.03180±0.00113a	0.02020±0.00148cd	0.00960±0.00074f
Isobutyl ketone	1207	*aldehydes and ketone*	0.00300±0.00006d	0.00480±0.00012bc	0.00380±0.00038cd	0.00710±0.00040a
Heptanal	1187	*aldehydes and ketone*	0.00060±0.00006e	0.00380±0.00020cd	0.00450±0.00095c	0.01770±0.00035a
trans-2-Hexenal	1329	*aldehydes and ketone*	0.00360±0.00013f	0.03420±0.00038a	0.02060±0.00198c	0.01270±0.00142e
2-Hexenal	1220	*aldehydes and ketone*	0.21820±0.00509f	1.45460±0.04182a	0.94960±0.10100c	0.79340±0.037550de
3-Octanone	1281	*aldehydes and ketone*	0.00020±0.00005d	0.00100±0.00017cd	0.00150±0.00037cd	0.02040±0.00139a
Octanal	1280	*aldehydes and ketone*	0.00050±0.00009c	0.00120±0.00013c	0.00170±0.00033c	0.01000±0.00138a
1-Octene-3-one	1438	*aldehydes and ketone*	0.00030±0.00002d	0.00500±0.00012bc	0.00540±0.00132b	0.00800±0.00048a
cis-2-Heptenal	1323	*aldehydes and ketone*	0.00030±0.00001e	0.01350±0.00024b	0.01030±0.00151c	0.02150±0.00086a
Sulcatone	1333	*aldehydes and ketone*	0.00040±0.00004f	0.00260±0.00016d	0.00340±0.00028c	0.00970±0.00059a
Nonanal	1386	*aldehydes and ketone*	0.00030±0.00002d	0.00050±0.00008cd	0.00050±0.00004cd	0.00130±0.00020a
Sorbaldehyde	1411	*aldehydes and ketone*	0.00280±0.00009f	0.01210±0.00020b	0.00780±0.00076d	0.00580±0.00021e
trans-2-Octenal	1428	*aldehydes and ketone*	0.00030±0.00003d	0.00530±0.00022c	0.00640±0.00136c	0.03540±0.00236a
trans-2-trans-4-Heptadienal	1494	*aldehydes and ketone*	0.00050±0.00002g	0.01520±0.00033a	0.00920±0.00058d	0.00940±0.00034d
Decanal	1504	*aldehydes and ketone*	0.00040±0.00004c	0.00050±0.00023c	0.00050±0.00014c	0.00170±0.00030a

Camphor	1516	*aldehydes and ketone*	0.00020±0.00001g	0.00100±0.00004c	0.00070±0.00007d	0.00110±0.00004b
trans-2-Nonenal	1679	*aldehydes and ketone*	0.00010±0.00002e	0.00140±0.00012c	0.00120±0.00036cd	0.01220±0.00091a
Cucumber aldehyde	1589	*aldehydes and ketone*	0.00010±0.00002e	0.00420±0.00023b	0.00280±0.00061c	0.01310±0.00095a
trans-2-Decenal	1206	*aldehydes and ketone*	0.00020±0.00001d	0.00050±0.00004bc	0.00050±0.00006cd	0.00230±0.00029a
trans, trans-2,4-Nonadienal	1703	*aldehydes and ketone*	—	0.00110±0.00011bc	0.00090±0.00009cd	0.00450±0.00026a
1-Pentene-3-ol	1162	*Isoprenoids*	0.00370±0.00020e	0.01080±0.00005a	0.00590±0.00091d	0.00700±0.00036cd
β-Myrcene	1170	*Isoprenoids*	—	—	—	—
D-Limonene	1203	*Isoprenoids*	—	—	0.00070±0.00008c	0.00590±0.00013a
β-Ocimene	1246	*Isoprenoids*	—	—	0.00040±0.00006c	0.00550±0.00012a
Dihydrolinalool	1255	*Isoprenoids*	0.00150±0.00046cde	0.00170±0.00036cd	0.00120±0.00008de	0.00500±0.00032a
cis-Linalool oxide	1441	*Isoprenoids*	—	0.00020±0.00002e	0.00050±0.00005d	0.00130±0.00006a
Morillol	1452	*Isoprenoids*	0.00490±0.00017f	0.04400±0.00131a	0.02250±0.00254d	0.03880±0.00100b
trans-Linalool oxide	1464	*Isoprenoids*	—	0.00070±0.00003d	0.00150±0.00023c	0.00400±0.00013a
Linalol	1527	*Isoprenoids*	0.00060±0.00006e	0.00450±0.00036e	0.03630±0.00550c	0.52800±0.00819a
Levomenthol	1651	*Isoprenoids*	0.00070±0.00018d	0.00090±0.00018cd	0.00070±0.00006d	0.00260±0.00012a
Farnesene	1749	*Isoprenoids*	—	—	0.00020±0.00006c	0.00640±0.00006a
α-Terpineol	1697	*Isoprenoids*	0.00010±0.00001c	0.00030±0.00005c	0.00100±0.00007c	0.01440±0.00155a
Bisabolene	1792	*Isoprenoids*	—	—	—	0.00100±0.00013a
Nerylacetone	1865	*Isoprenoids*	0.0001±0.00004bc	0.0003±0.00003bc	0.00040±0.00007b	0.00200±0.00039a
Bisabolol	2207	*Isoprenoids*	—	—	—	0.00180±0.00063a
β-Damascenone	1808	*Isoprenoids*	0.00030±0.00005b	0.00710±0.00057a	0.00470±0.00036a	0.00610±0.00054a
Nerolidol	1961	*Isoprenoids*	—	—	0.00100±0.00029b	0.14740±0.01521a
1-p-Menthen-9-al	1212	*Isoprenoids*	0.0000±0.00001e	0.00070±0.00005d	0.00120±0.00004c	0.00580±0.00031a
2-Pentylfurane	1231	*furan*	0.00040±0.00002c	0.00650±0.00100bc	0.00460±0.00073bc	0.06460±0.00455a
Furfural	1472	*furan*	0.00140±0.00008c	0.0002±0.00001e	0.0002±0.00002e	0.00070±0.00006d

Note:different letters represent comparison of the same volatile components in four periods of 'Benihoppe' (WT, Table S2) and somaclonal mutant 'Xiaobai' (*mut*, Table S3)

Table S4. The proportion of nine categories of volatile compounds in four developmental stages of fruit between 'Benihoppe' (WT) and somaclonal mutant 'Xiaobai' (*mut*).

	**WT-Green**	**WT-White**	**WT-Turn**	**WT-Red**	***mut*-Green**	***mut*-White**	***mut*-Turn**	***mut*-Red**
Alcohols	8.21%	3.34%	2.38%	2.33%	11.67%	3.07%	2.92%	0.56%
Acid	0.97%	0.44%	2.43%	26.18%	2.97%	0.66%	9.06%	16.61%
esters	0.85%	1.44%	8.20%	32.24%	4.31%	3.38%	15.82%	70.45%
Benzene and volatile phenols	1.17%	1.05%	1.00%	1.05%	2.27%	0.85%	1.37%	1.34%
aldehydes and ketone	87.01%	90.60%	82.86%	33.74%	75.67%	89.04%	67.34%	7.52%
Isoprenoids	1.50%	2.68%	2.79%	4.23%	2.71%	2.73%	3.28%	3.26%
furan	0.30%	0.44%	0.34%	0.21%	0.41%	0.26%	0.20%	0.27%

（The esters in Table S4 included the ethyl esters, acetates, and other esters)

Table S5. The ratio of volatile substances contents in 'Benihoppe' (WT) and somaclonal mutant 'Xiaobai' (*mut*) in four developmental stages.

	**Green mut/WT**	**White mut/WT**	**Turn mut/WT**	**Red mut/WT**
Alcohols	0.25	1.83	1.10	1.12
Acid	0.54	2.98	3.33	2.96
Ethyl esters	0.57	2.40	3.55	56.57
acetate esters	1.05	4.75	1.24	7.29
other esters	0.38	5.81	4.99	1.23
Benzene and volatile phenols	0.34	1.59	1.23	5.93
aldehydes and ketone	0.15	1.96	0.73	1.04
Isoprenoids	0.32	2.03	1.05	3.60
furan	0.24	1.18	0.52	5.99

Table S6. The unique volatile components in 'Benihoppe' (WT).

**Period**	**The volatile components**	***classification***
Green	Methyl acetate	*acetate esters*
	Hexyl acetate	*acetate esters*
	trans-2-Hexenyl acetate	*acetate esters*
	trans, trans-2,4-Nonadienal	*aldehydes and ketone*
	cis-Linalol oxide	*Isoprenoids*
	trans-Linalool oxide (furanoid)	*Isoprenoids*
	Farnesene	*Isoprenoids*
White	Methoxypropanol	*Alcohols*
	Isobutyric acid	*Acid*
	Heptylic acid	*Acid*
Turn	trans-2-Hexenyl Butyrate	*other esters*
Red	Pentyl alcohol	*Alcohols*

Table S7. The unique volatile components in somaclonal mutant 'Xiaobai' (*mut*).

**Period**	**The aroma components**	***classification***
Green	2-methyl-5-Hepten-1-ol	*Alcohols*
	Ethyl pentanoate	*Ethyl esters*
	Ethyl benzoate	*Benzene and volatile phenols*
	Benzyl acetate	*Benzene and volatile phenols*
	Cinnamyl acetate	*Benzene and volatile phenols*
	Nerylacetone	*Isoprenoids*
White	2-methyl-5-Hepten-1-ol	*Alcohols*
	Ethyl pentanoate	*Ethyl esters*
	Methyl butyrate	*other esters*
	Benzyl acetate	*Benzene and volatile phenols*
Turn	Methoxypropanol	*Alcohols*
	2-methyl-5-Hepten-1-ol	*Alcohols*
	trans-3-Hexenoic Acid	*Acid*
	trans-2-Octenoic acid	*Acid*
	Ethyl isovalerate	*Ethyl esters*
	Ethyl pentanoate	*Ethyl esters*
	isopentenyl acetate	*acetate esters*
	Isoamyl butylate	*other esters*
	Octyl isovalerate	*other esters*
	Benzeneacetaldehyde	*Benzene and volatile phenols*
	Eugenol	*Benzene and volatile phenols*
	Farnesene	*Isoprenoids*
	Nerolidol	*Isoprenoids*
Red	Methoxypropanol	*Alcohols*

trans-2-Octenoic acid	*Acid*
Ethyl 2-hexenoate	*Ethyl esters*
Ethyl 3-hydroxyhexanoate	*Ethyl esters*
Propyl isovalerate	*other esters*
Octyl isovalerate	*other esters*
Benzeneacet aldehyde	*Benzene and volatile phenols*
Ethyl benzoate	*Benzene and volatile phenols*
Cinnamyl acetate	*Benzene and volatile phenols*
Bisabolene	*Isoprenoids*
Nerylacetone	*Isoprenoids*
Bisabolol	*Isoprenoids*

Table S8. Principal component load matrix of volatile compounds.

**Categories**	**PC1**	**PC2**
Alcohols	0.14890665	-0.65112885
Acid	0.37975818	0.06789272
Ethyl esters	0.37326131	0.09826685
Acetate esters	0.38004148	0.08590762
Other esters	0.33377753	0.02145567
Benzene and volatile phenols	0.38012223	0.04543039
Aldehydes and.ketone	0.06851612	-0.74088228
Isoprenoids	0.38290603	0.04746335
Furan	0.37768129	0.02649001
